# Cardiac Imaging and Individual Risk Assessment: Current State-of-the-Art and Economical Implications

**DOI:** 10.5334/jbr-btr.1098

**Published:** 2016-04-11

**Authors:** Tim Leiner

**Affiliations:** 1 UMC Utrecht, NL

Cardiac magnetic resonance (CMR) imaging is a widely used diagnostic tool in clinical practice. Besides diagnostic information, CMR may also provide prognostic information for future cardiovascular events in patients with a recent myocardial infarction (MI) and patients with suspected or known coronary artery disease (CAD).

In this lecture, the diagnostic value of CMR findings is established and the independent prognostic association with future cardiovascular events is explored. The lecture is based on a meta-analysis and systematic review of prognostic studies identified by systematic MEDLINE and EMBASE searches on this topic. Published literature is reviewed for associations between CMR findings (left ventricular ejection fraction [LVEF], wall motion abnormalities [WMA], abnormal myocardial perfusion, microvascular obstruction, late gadolinium enhancement, edema, and intramyocardial hemorrhage) and hard events (all-cause mortality, cardiac death, cardiac transplantation, and MI) or major adverse cardiovascular events (MACE) (hard events and other cardiovascular events defined by the authors of the evaluated papers).

Fifty-six studies (n = 25,497) were evaluated. For patients with recent MI, too few patients were evaluated to establish associations between CMR findings and hard events. LVEF (range of adjusted hazard ratios [HRs]: 1.03 to 1.05 per % decrease) was independently associated with MACE. In patients with suspected or known CAD, WMA (adjusted HRs: 1.87 to 2.99); inducible perfusion defects (adjusted HRs: 3.02 to 7.77); LVEF (adjusted HRs: 0.72 to 0.82 per 10% increase); and infarction (adjusted HRs: 2.82 to 9.43) were independently associated with hard events, and the presence of inducible perfusion defects was associated with MACE (adjusted HRs: 1.76 to 3.21). The independent predictor of future cardiovascular events for patients with a recent MI was LVEF, and the predictors for patients with suspected or known CAD were WMA, inducible perfusion defects, LVEF, and presence of infarction.

## Competing Interests

The author declares that they have no competing interests.

